# Acta Veterinaria Scandinavica reviewer acknowledgement 2013

**DOI:** 10.1186/1751-0147-56-15

**Published:** 2014-05-26

**Authors:** Jørgen S Agerholm

**Affiliations:** 1 Department of Large Animal Sciences, Faculty of Health and Medical Sciences, University of Copenhagen, Dyrlægevej 68, DK-1870 Frederiksberg C, Denmark

## Abstract

**Contributing reviewers:**

The *Acta Veterinaria Scandinavica* editorial team would like to thank the following colleagues who contributed to peer review for the journal in 2013.

## 

Luis Calvinho

Argentina

David Evans

Australia

Frank Nicholas

Australia

Nerissa Stander

Australia

Michael J Studdert

Australia

Martina Flöck

Austria

Michaela Gumpenberger

Austria

Ernst Leidinger

Austria

Christoph Winckler

Austria

Ahmad Rabiee

Azerbaijan

Christian Burvenich

Belgium

Undine Christmann

Belgium

Jeroen Dewulf

Belgium

Maarten Hoogewijs

Belgium

Dominiek Maes

Belgium

Davy Persoons

Belgium

Bart Van Goethem

Belgium

Maira Souza Oliveira

Brazil

Carlos Termignoni

Brazil

Sebastien Buczinski

Canada

Etienne Côté

Canada

José Denis-Robichaud

Canada

A Hossain Farid

Canada

Martin Guillot

Canada

Janet Hill

Canada

Stephen LeBlanc

Canada

Kathleen Linn

Canada

Christopher Luby

Canada

Jérôme Planté

Canada

Scott Weese

Canada

Klas Abelson

Denmark

Jens Agger

Denmark

Aage Alstrup

Denmark

Mads Bertelsen

Denmark

Peter Bollen

Denmark

Rikke Buhl

Denmark

Henrik Callesen

Denmark

Henrik Christensen

Denmark

Charlotte Maria Dalsgaard

Denmark

Birthe Damgaard

Denmark

Jörg Matthias Dehn Enemark

Denmark

Rikke Fast

Denmark

Anne Sofie Hammer

Denmark

Anne-Marie Heegaard

Denmark

Mette Herskin

Denmark

Stine Jacobsen

Denmark

Henrik Elvang Jensen

Denmark

Anders Ringgaard Kristensen

Denmark

Anders Larsen

Denmark

Nanna Luthersson

Denmark

Jens Malmkvist

Denmark

Line Morgills

Denmark

Ole Lerberg Nielsen

Denmark

Lisbeth Høier Olsen

Denmark

Karl Pedersen

Denmark

Mette Schmidt

Denmark

Paul E Simonsen

Denmark

Mette Tingleff Skaanild

Denmark

Tine Søland

Denmark

Dorte Bratbo Sørensen

Denmark

Pernille Tveden-Nyborg

Denmark

Håkan Vigre

Denmark

Brian Lassen

Estonia

Toomas Orro

Estonia

Arvo Viltrop

Estonia

Andres Waldmann

Estonia

Magnus Carl Andersson

Finland

Marjukka Anttila

Finland

Laura Hänninen

Finland

Marja-Liisa Hänninen

Finland

Mari Heinonen

Finland

Udo Hetzel

Finland

Anita Huovilainen

Finland

Liisa Kaartinen

Finland

Paula M Kinnunen

Finland

Outi Laitinen-Vapaavuori

Finland

Anna-Liisa Myllyniemi

Finland

Mauri Nieminen

Finland

Raimo Pohjanvirta

Finland

Satu Pyörälä

Finland

Merja Rantala

Finland

Helena Rautala

Finland

Liisa Helena Sihvonen

Finland

Juhani Taponen

Finland

Suvi Taponen

Finland

Riitta-Mari Tulamo

Finland

Pirkko Lucia Tuominen

Finland

Pekka Uimari

Finland

François Casas

France

Gilles Charpigny

France

Xavier Druart

France

Gilles Foucras

France

Lucia Manso-Silvan

France

Emmanuelle Robardet

France

Sally Solomon

France

Francois Thiaucourt

France

Berit Bangoura

Germany

Armando Damiani

Germany

Viktor Dyachenko

Germany

Manfred Kietzmann

Germany

Wolfgang Klee

Germany

Stefanie Klenner

Germany

Robert Klopfleisch

Germany

Klaus Henning

Germany

Wolfgang Holtz

Germany

Arno Lindner

Germany

Burkhard Malorny

Germany

Reinhard Mischke

Germany

Karin Müller

Germany

Ralf Müller

Germany

Heinrich Neubauer

Germany

Gerald Rimbach

Germany

Hans-Joachim Schuberth

Germany

Gabriele Stangl

Germany

Michael Wendt

Germany

Wilhelm Windisch

Germany

Ilias Giannenas

Greece

Tomas Guerrero

Grenada

Szabolcs Nagy

Hungary

Grétar Harðarson

Iceland

Lena Englund

Ireland

Itamar Aroch

Israel

Mark Pines

Israel

Tiziana Bellini

Italy

Nicola Decaro

Italy

Luigi Calamari

Italy

Angela Di Cesare

Italy

Gaetano Mari

Italy

Paolo Pasquali

Italy

Alessandro Poli

Italy

Monica Probo

Italy

Tetsuo Asai

Japan

Junko Kohara

Japan

Makoto Miyata

Japan

Susanne Boroffka

Netherlands

Menno Holzhauer

Netherlands

Paul Mandigers

Netherlands

Auke Schaefers-Okkens

Netherlands

Viktor Szatmári

Netherlands

Cornelis van Elk

Netherlands

Frederik J van Sluijs

Netherlands

Jon Hickford

New Zealand

Keith Thompson

New Zealand

Jon M Arnemo

Norway

Nils Ivar Dolvik

Norway

Arild Espenes

Norway

Terje Fjeldaas

Norway

Tore Framstad

Norway

Anna Germundsson

Norway

Karin Hultin Jäderlund

Norway

Carl Fredrik Ihler

Norway

Paal Johnsen

Norway

Hannah Joan Jørgensen

Norway

Randi Ingebjørg Krontveit

Norway

Thor Landsverk

Norway

Bjørn Lium

Norway

Tormod Mork

Norway

Stephen Mutoloki

Norway

Liv Torunn Mydland

Norway

Anne Berit Olsen

Norway

Liv Jorun Reitan

Norway

Bente Kristin Sævik

Norway

Tore Sivertsen

Norway

Ellen Skancke

Norway

Eystein Skjerve

Norway

Hege Kippenes Skogmo

Norway

Snorre Stuen

Norway

Synnøve Vatn

Norway

Arne Yndestad

Norway

Malgorzata Brzóska

Poland

Tadeusz Frymus

Poland

Jacek Gajek

Poland

Urszula Paslawska

Poland

Agata Piestrzynska-Kajtoch

Poland

Piotr Szefer

Poland

Dariusz Wasyl

Poland

Tomasz Wielkoszynski

PolandPoland

Fatima Gartner

Portugal

Peter Massanyi

Slovakia

Dusan Bencina

Slovenia

Marjeta Candek-Potokar

Slovenia

Martie van der Walt

South Africa

Sonia Almeria

Spain

Librado Carrasco

Spain

Pia Haubro Andersen

Sweden

Eva Axnér

Sweden

Renée Båge

Sweden

Viveca Båverud

Sweden

Bjorn Bengtsson

Sweden

Anna Bergh

Sweden

Kerstin Bergvall

Sweden

Orjan Carlborg

Sweden

Agneta Egenvall

Sweden

Stig Einarsson

Sweden

Stina Ekman

Sweden

Marianne Elvander

Sweden

Helle Ericsson Unnerstad

Sweden

Ingrid Hansson

Sweden

Eva Hellmén

Sweden

Odd Viking Hoglund

Sweden

Kjell Holtenius

Sweden

Magdalena Jacobson

Sweden

Anna Jansson

Sweden

Hans Kindahl

Sweden

Leif Kirsebom

Sweden

Anne-Sofie Lagerstedt

Sweden

Charles Ley

Sweden

Cecilia Rohdin

Sweden

Bengt Röken

Sweden

Bodil Strom Holst

Sweden

Anna Tidholm

Sweden

Per Wallgren

Sweden

Manja Zupan

Sweden

Valentino Cattori

Switzerland

Mark Flückiger

Switzerland

Paola Gandolfi-Decristophoris

Switzerland

David Spreng

Switzerland

Adrian Steiner

Switzerland

Monika Welle

Switzerland

Mark Bowen

United Kingdom

Marnie Brennan

United Kingdom

Lucy Brunton

United Kingdom

Harry Carslake

United Kingdom

Fiona Cooke

United Kingdom

Robert Davies

United Kingdom

Steven De Decker

United Kingdom

Hilary Dobson

United Kingdom

Laura Green

United Kingdom

Edward Hall

United Kingdom

Katherine Hughes

United Kingdom

Jolyon Medlock

United Kingdom

Susan Paterson

United Kingdom

Simon Priestnall

United Kingdom

Tobias Schwarz

United Kingdom

Phil Scott

United Kingdom

Jenny Stavisky

United Kingdom

Frank Andrews

United States of America

Paul Bartlett

United States of America

Alvin Camus

United States of America

Thomas Casey

United States of America

Roger Clemmons

United States of America

Hans Coetzee

United States of America

James Evermann

United States of America

John C Godbold Jr

United States of America

Barbara Gores

United States of America

John Griffin

United States of America

Rita Hanel

United States of America

Steve Hodges

United States of America

Michele Jay-Russell

United States of America

Mark Jenkins

United States of America

Anil Kadegowda

United States of America

Katherine Kocan

United States of America

Bradley Kropp

United States of America

Michelle Kutzler

United States of America

Paulraj Lawrence

United States of America

Jane Manfredi

United States of America

Stanley Marks

United States of America

Mark Mitchell

United States of America

Tiruvoor Nagaraja

United States of America

Stephen Nickerson

United States of America

Annette O'Connor

United States of America

Robert Paine

United States of America

Elena Alina Palade

United States of America

Mary Pantin-Jackwood

United States of America

Narda Robinson

United States of America

Subramaniam Srikumaran

United States of America

Olivier Taeymans

United States of America

John Timoney

United States of America

Betsy Vaughan

United States of America

Deborah Vela

United States of America

Robert Washabau

United States of America

Robert Weedon

United States of America

Zheng Xing

United States of America

Laszlo Zsak

United States of America

Alejo Menchaca

Uruguay

